# Growing in the Shadow of Mental Illness: Quality of Life, Health Status, Social Functioning, and Health Care Utilization Among Adults to Parents With Severe Mental Illness

**DOI:** 10.1111/jnu.70118

**Published:** 2026-07-26

**Authors:** Alexander Shestiperov, Ilya Kagan, Orli Grinstein‐Cohen

**Affiliations:** ^1^ Department of Nursing, Faculty of Health Sciences Ben‐Gurion University of the Negev Beer‐Sheva Israel; ^2^ Merhavim‐Mental Health Center Beer‐Yaakov Israel; ^3^ Nursing Department Ashkelon Academic College Ashkelon Israel

**Keywords:** childhood trauma, mental health, parental bonding, resilience, self‐efficacy, SMI

## Abstract

**Introduction:**

Adults who grow up with a parent with severe mental illness are exposed to prolonged developmental adversity, yet little is known about how this experience shapes multidimensional outcomes in adulthood. In particular, the roles of personal adaptive resources, childhood trauma, parental bonding, and morbidity in influencing quality of life, social functioning, and healthcare utilization remain insufficiently understood. This study aimed to examine the relationships between personal adaptive resources (resilience and general self‐efficacy), childhood experiences (trauma and parental bonding), and adult outcomes, including quality of life, social functioning, morbidity, and healthcare utilization, as well as to examine the moderating role of resilience.

**Design:**

A cross‐sectional quantitative design was employed.

**Methods:**

A convenience sample of 300 adults who grew up with a parent with severe mental illness completed a self‐report questionnaire which consisted of validated scales for study variables: Personal Wellbeing Index‐Adult, Connor‐Davidson Resilience Scale‐10, New General Self‐Efficacy Scale, Social Functioning Questionnaire, Parental Bonding Instrument, Childhood Trauma Questionnaire, and EUROHIS survey items for morbidity and healthcare utilization. Data were analyzed using Spearman correlations, hierarchical multiple linear regression, negative binomial regression, and moderation analysis using PROCESS.

**Results:**

Resilience and self‐efficacy independently predicted higher quality of life. Childhood trauma and parental overprotection independently predicted poorer social functioning, while parental care did not contribute unique variance in multivariate models. Childhood trauma significantly predicted morbidity, and morbidity was associated with increased healthcare utilization. Resilience did not moderate the relationship between childhood trauma and either morbidity or social functioning, indicating independent rather than interactive effects.

**Conclusion:**

Among adults who grew up with a parent with SMI, resilience and self‐efficacy function as independent contributors to subjective wellbeing, while childhood adversity and morbidity shape long‐term social and health outcomes. Resilience does not appear to buffer the long‐term effects of trauma on health or functioning.

## 1 Introduction

Worldwide, an estimated 12%–23% of children grow up in a household where at least one parent has a mental illness (Grant et al. 2024; Maybery and Reupert 2018). When that illness is severe, characterized by persistent functional impairment requiring repeated psychiatric hospitalization, the consequences for the child extend far beyond childhood, associated with a developmental trajectory marked by chronic adversity, disrupted attachment, and pervasive psychosocial risk (Brummelhuis et al. 2025; Harries et al. 2023). Despite the scale of this public health reality, adults who grew up with a severely mentally ill [SMI] parent represent one of the most systematically overlooked populations in mental health research.

SMI remains without universal consensus in its definition worldwide. While the National Institute of Mental Health defines SMI as any condition causing significant functional impairment that substantially interferes with essential life activities (NIMH 2023), other frameworks emphasize chronicity and service dependence as defining features (Martínez‐Martínez et al. 2020). Globally, an estimated one in eight people live with a mental disorder, with SMI accounting for a disproportionate share of associated disability and premature mortality (WHO 2025). No multidimensional quantitative study has examined how the cumulative burden of this childhood experience is associated with health, functioning, and quality of life in adulthood, the period when these individuals are building careers, forming families, and navigating healthcare systems (Brummelhuis et al. 2025; Shestiperov et al. 2024).

Parental SMI disrupts caregiving across every developmental stage, constituting not a discrete adverse event but a sustained environmental condition. In infancy, affective unavailability and disrupted primary bonding can progress to neglect and developmental delay. During the school years, impaired parental communication and inconsistent limit‐setting are associated with compromised behavioral and academic development (Harries et al. 2023). In adolescence, the effects extend to social functioning, decision‐making, and identity formation, frequently accompanied by role reversal, a dynamic in which the child assumes responsibility for the ill parent's basic needs, finances, and care (Harries et al. 2023; Shestiperov et al. 2024). These processes are compounded by direct exposure to traumatic experiences, including physical and verbal violence, material neglect, and recurrent psychiatric crises, which are further associated with elevated risk across the biopsychosocial spectrum (Kerbage and Purper‐Ouakil 2024; Visser et al. 2026; Witt et al. 2019).

The adult consequences of growing up under these conditions are only beginning to come into focus. In a sample of 59 adults who grew up with a mentally ill parent, 89.9% reported current or historical psychological problems, 94.9% experienced interpersonal difficulties, and 89.1% had regular physical symptoms, yet in more than half of those who sought professional help, their background was never acknowledged or addressed in treatment (Brummelhuis et al. 2025). The risk of developing a mental disorder in adulthood has been estimated at up to 13‐fold above the general population (Dean et al. 2010), with more recent findings revealing increased risks specifically for anxiety, depression, suicidality, somatoform disorders, and substance abuse (Brummelhuis et al. 2022), while the broader literature consistently documents lower quality of life, impaired psychosocial functioning, and educational and employment disadvantage in this group. While evidence‐based interventions have been developed for children and young people with a parent with mental illness, most target those under 18 years, and the evidence base remains emergent (Lannes et al. 2021; Reupert et al. 2022). A small number of programs address young adults aged 18–25 years who have a parent with a mental illness, including online interventions showing preliminary efficacy (Maybery et al. 2022), and recent Delphi work has begun to identify support needs specifically among adult children (Patrick et al. 2023). However, no interventions have been developed or evaluated specifically for adults who grew up with a parent with SMI. This population is defined by the severity and chronicity of parental illness rather than its presence alone, and the implementation of existing programs into routine clinical services remains a critical unresolved challenge (Reupert et al. 2022; Barnardos 2024). The specific pathways through which childhood adversity is associated with adult health outcomes in this population remain uncharted (Brummelhuis et al. 2025).

The established literature provides a framework for these pathways, though none has been tested in this specific population. General self‐efficacy, the belief in one's capacity to function and achieve across diverse challenging situations, is consistently associated with better quality of life, psychological wellbeing, and adaptive coping (Bonsaksen et al. 2019; FitzGerald et al. 2022; Sharma 2023). Personal resilience is consistently associated with better physical and mental health outcomes and psychosocial functioning (Seiler and Jenewein 2019). Childhood traumatic experiences are robustly linked to morbidity, impaired social functioning, and greater healthcare service utilization in adulthood (Hailes et al. 2019; Karatzias et al. 2020; Merrick et al. 2017). The quality of the parent–child bond adds a further dimension: overprotection and low parental care are associated with poorer mental health and reduced social functioning in later life (Kullberg et al. 2020; Thanhäuser et al. 2017; Wiegand‐Grefe et al. 2019). Whether and how these pathways intersect in adults who grew up specifically with an SMI parent has not been examined.

This study therefore aimed to address these gaps through a cross‐sectional quantitative investigation of adults who grew up with an SMI parent. Specifically, the study examined: (1) the associations between general self‐efficacy, resilience, and quality of life; (2) the relationships between childhood traumatic experiences and parental bonding and their associations with physical and mental morbidity and social functioning; (3) the associations between morbidity and quality of life and healthcare service utilization; (4) the moderating role of resilience on the relationships between childhood trauma and both morbidity and social functioning.

## 2 Design

A cross‐sectional design was employed to examine the relationships between personal adaptive resources (resilience and general self‐efficacy), childhood adversity (including traumatic experiences and parental bonding), and key adult outcomes, including quality of life, health status, social functioning, and healthcare service utilization, among adults who grew up with a parent with SMI. The present study constitutes the quantitative phase of a sequential mixed‐methods research program and builds directly on a prior interpretive phenomenological study conducted with 30 adults in Israel who grew up with a parent with SMI (Shestiperov et al. 2024). That study identified four main themes within an overarching theme of “Childhood survival into adulthood survival”: traumatic childhood, characterized by neglect, parentification, role reversal, and exposure to violence; perceived control, describing adaptive coping developed under chronic parental dysfunction; resilience and general self‐efficacy, referring to enduring orientations of self‐reliance and confidence that consolidated during the transition to adulthood; and adult quality of life, marked by a paradoxical coexistence of social and occupational achievement alongside pervasive loneliness, caregiving burden, and avoidance of help‐seeking. A key finding was that the adaptive capacities enabling functioning of resilience and self‐efficacy simultaneously generated costs, including persistent reluctance to seek professional help. These qualitative findings directly informed the present study's selection of variables, choice of instruments, and specification of research aims. Participants from the qualitative study did not take part in the present quantitative study, ensuring the independence of the two samples.

## 3 Materials and Methods

### 3.1 Procedure

Following institutional and ethical approvals, participants were recruited through a public call posted on Facebook groups for individuals with mental illness and family members of people with mental illness, in community centers and regional family support centers, and across psychiatric facilities including community‐care clinics, emergency departments, and inpatient and outpatient units throughout Israel. During scheduled family visiting hours at inpatient psychiatric units, the primary researcher directly approached potential participants. Individuals who expressed interest contacted the researcher by phone, email, or instant message to confirm eligibility. Those who met the inclusion criteria were scheduled for a meeting at a time and location of their choosing.

At the meeting, participants received a full explanation of the study, reviewed the informed consent form, and were encouraged to ask questions before, during, or after questionnaire completion. All participants provided written informed consent prior to enrollment. Each eligible participant received a printed, self‐administered questionnaire, completed it during the session, and returned it to the researcher before leaving.

### 3.2 Participants

Participants were Hebrew‐speaking adults (aged ≥ 18 years) residing in Israel who had grown up before the age of 18 with a parent with SMI. While there is no consensus regarding a global definition of SMI (Gonzales et al. 2022), this study operationalized parental SMI using a two‐dimensional criterion based on participant self‐report: (a) a formal psychiatric diagnosis accompanied by (b) at least two psychiatric hospitalizations during the participant's childhood. Individuals were excluded if cognitive or physical impairments precluded independent questionnaire completion or if they had a legal guardian.

Participants were recruited using a convenience sampling strategy with snowball recruitment, consistent with methodological recommendations for research involving sensitive and hard‐to‐reach populations (Hilário et al. 2023; Raifman et al. 2017; Valerio et al. 2016). To reduce recruitment bias, sampling was deliberately extended beyond psychiatric settings to include community centers, regional family support centers, and social media platforms (Raifman et al. 2017).

The sample comprised 300 adults who grew up with a parent diagnosed with SMI, aged 18–62 years (*M* = 36.33, SD = 8.82). Data were collected between August 2023 and January 2026. Of the participants, 161 (53.7%) were female; 194 (64.7%) were married or in a partnership; 233 (77.7%) were Israeli‐born; and 224 (74.7%) were employed at the time of data collection (Table 1).

**TABLE 1:** Participant characteristics (*N* = 300).

Variables	*n* (%)	*M* (SD)
Sociodemographic		
Age (years)	—	36.33 (8.82)
Years of formal education	—	15.31 (2.90)
Number of children	—	1.77 (1.56)
Number of siblings	—	2.44 (1.86)
Gender		
Female	161 (53.7%)	—
Male	139 (46.3%)	—
Marital status		
Married	194 (64.7%)	—
Single	83 (27.7%)	—
Divorced	21 (7.0%)	—
Other	2 (0.7%)	—
Employment status		
Employee	224 (74.7%)	—
Self‐employed	42 (14.0%)	—
Other	18 (6.0%)	—
Unemployed	9 (3.0%)	—
Pensioner	7 (2.3%)	—
Country of birth		
Israel	233 (77.7%)	—
Other	67 (22.3%)	—
Parent with SMI		
Biological mother	195 (65.0%)	—
Biological father	98 (32.7%)	—
Stepfather/foster father	4 (1.3%)	—
Stepmother/foster mother	3 (1.0%)	—
Birth order among siblings		
First‐born	139 (46.3%)	—
Second‐born	85 (28.3%)	—
Third‐born	39 (13.0%)	—
Fourth‐born or later (4–7)	37 (12.3%)	—
Frequency of financial problems (past year)		
Very rarely	167 (55.7%)	—
Rarely	81 (27.0%)	—
Often	36 (12.0%)	—
Very often	16 (5.3%)	—
Financial and health self‐ratings		
Self‐rated financial situation (1–10)	—	7.59 (2.11)
Self‐rated current health (1–10)	—	8.37 (1.83)

In terms of educational and family characteristics, participants reported a mean of 15.31 years of formal education (SD = 2.90); the mean number of children was 1.77 (SD = 1.56), and the mean number of siblings was 2.44 (SD = 1.86). First‐born children comprised the largest birth order group (*n* = 139, 46.3%). Regarding financial status, participants' mean self‐rated financial situation was 7.59 out of 10 (SD = 2.11), and 167 participants (55.7%) reported experiencing financial difficulties very rarely in the past year. Mean self‐rated current health was 8.37 out of 10 (SD = 1.83; Table 1).

Regarding the affected parent, the majority of participants grew up with a mother diagnosed with SMI (*n* = 198, 66%), with the remainder reporting a father as the affected parent (*n* = 102, 34%; Table 1).

### 3.3 Sample Size

An a priori sample size calculation was conducted using G*Power 3.1 (Faul et al. 2007) for multiple linear regression (fixed model). Assuming 80% statistical power (1‐*β* = 0.80), a two‐tailed significance level of *α* = 0.05, a medium effect size (*f* ^2^ = 0.15; Cohen, 1988), and 15 predictors in the largest planned model, a minimum sample of 139 participants was required. The final sample of 300 participants exceeded this requirement, providing sufficient statistical power for the planned analyses.

### 3.4 Ethical Considerations

The study was conducted following the approval of the institutional review boards of Ben‐Gurion University of the Negev (#45‐2022) and the Barzilai Medical Center (#BRZ‐0099‐22). According to the ethical approvals and study protocol, to address the emotional sensitivity of the study topic, participants were provided with access to 24/7 mental health support resources and the direct contact details of the primary researcher, a licensed mental health nurse specialist, available for immediate support if needed.

### 3.5 Data Collection

Following eligibility confirmation and informed consent, participants completed a structured, self‐administered paper questionnaire. The questionnaire was developed and refined on the basis of findings from a prior qualitative study (Shestiperov et al. 2024) and included instruments according to study aims. All instruments were used with formal permissions and legal authorizations obtained from the original authors where required; remaining instruments were publicly available for research use.

### 3.6 Instruments

*Quality of life* was assessed using the Personal Wellbeing Index‐Adult [PWI‐A] (International Wellbeing Group 2013), a widely used measure of subjective wellbeing comprising seven core items evaluating satisfaction across domains of standard of living, health, life achievements, interpersonal relationships, personal safety, community belonging, and future security. Each item is rated on an 11‐point scale (0 = no satisfaction at all, 10 = completely satisfied). The composite score is the mean of the seven items multiplied by 10, yielding a range of 0–100, with higher scores reflecting greater quality of life. The PWI‐A has demonstrated good internal consistency in previous research (*α* = 0.78–0.85; International Wellbeing Group 2013). In the present study, internal consistency was high (*α* = 0.925).

*Resilience* was assessed using the Connor‐Davidson Resilience Scale‐10 [CD‐RISC‐10] (Campbell‐Sills and Stein 2007), a 10‐item measure of personal resilience. Items are rated on a 5‐point scale (0 = not true at all, 4 = true nearly all of the time), and the composite score is the sum of all items, yielding a range of 0–40, with higher scores reflecting greater resilience. The CD‐RISC‐10 has demonstrated strong internal consistency in prior studies (*α* = 0.91; Kimhi et al. 2020). In the present study, internal consistency was high (*α* = 0.941).

*General self‐efficacy* was assessed using the New General Self‐Efficacy Scale [NGSE] (Chen et al. 2001), an 8‐item measure evaluating one's overall belief in one's ability to perform successfully across a variety of life events. Items are rated on a 5‐point scale (1 = strongly disagree, 5 = strongly agree), and the composite score is the mean of all items, with higher scores indicating greater self‐efficacy. The NGSE has demonstrated strong internal consistency in previous research (*α* = 0.90; Peled and Brunstein‐Klomek 2019). In the present study, internal consistency was high (*α* = 0.941).

*Childhood trauma exposure* was assessed using the Childhood Trauma Questionnaire‐Short Form [CTQ‐SF] (Bernstein et al. 1994), a 28‐item retrospective self‐report instrument measuring the presence and severity of childhood maltreatment across five subscales: Emotional Abuse, Physical Abuse, Sexual Abuse, Emotional Neglect, and Physical Neglect. Items are rated on a 5‐point frequency scale (1 = never true, 5 = very often true), and each subscale score is the sum of its five items, yielding a range of 5–25 per subscale. The total score of trauma burden is the sum of all subscales. Higher scores indicate greater trauma severity. The CTQ‐SF has demonstrated good internal consistency across subscales in previous research (*α* = 0.79–0.94; Bernstein et al. 1994; Herscu 2015; Tamarkin 2007). In the present study, internal consistency across subscales ranged from *α* = 0.767–0.892. The Sexual Abuse subscale demonstrated near‐zero variance in the current sample (skewness = 11.57) and was therefore not included in inferential analyses. Descriptive statistics for this subscale are presented in Table 2 and its bivariate associations are reported in Table 3 for transparency.

**TABLE 2:** Descriptive statistics and normality tests for all study variables (*N* = 300).

Variable	*M*	SD	Min	Max	Skewness	Kurtosis	SW (*p*)
Quality of Life (PWI‐A)	75.44	18.46	8.57	100.00	−1.189	1.186	< 0.001
Resilience (CD‐RISC‐10)	31.56	8.09	4.00	40.00	−1.210	0.980	< 0.001
General Self‐Efficacy (NGSE)	4.28	0.76	1.00	5.00	−1.379	1.856	< 0.001
Childhood Trauma (CTQ‐SF)	41.77	14.16	25.00	101.00	1.389	1.475	< 0.001
Emotional Abuse	8.04	4.16	5.00	25.00	1.876	3.313	< 0.001
Physical Abuse	5.96	2.72	5.00	25.00	3.299	10.543	< 0.001
Sexual Abuse^a^	5.11	0.82	5.00	17.00	11.565	155.167	< 0.001
Emotional Neglect	12.52	5.27	5.00	25.00	−0.004	0.270	< 0.001
Physical Neglect	10.14	4.16	5.00	24.00	0.904	0.452	< 0.001
Social Functioning (SFQ)	5.70	4.68	0.00	21.00	1.041	0.650	< 0.001
Parental Bonding (PBI)							
Care subscale	25.18	9.58	0.00	36.00	−0.835	−0.328	< 0.001
Overprotection subscale	9.38	8.57	0.00	38.00	1.022	0.334	< 0.001
Morbidity	1.39	1.94	0.00	10.00	2.100	4.659	< 0.001
Healthcare Utilization	2.12	2.84	0.00	17.00	2.452	7.559	< 0.001

*Note:* All Shapiro–Wilk tests significant at *p* < 0.001, indicating violation of normality. Spearman rank‐order correlations were used in all bivariate analyses accordingly.

^a^ Sexual Abuse subscale reported for descriptive purposes only.

**TABLE 3:** Spearman rank‐order correlations among all study variables (*N* = 300).

Variable	1	2	3	4	5	6	7	8	9	10	11	12	13	14
1. Quality of Life														
2. Resilience	0.693***													
3. General Self‐Efficacy	0.651***	0.840***												
4. Social Functioning	−0.666***	−0.646***	−0.654***											
5. Emotional Abuse (CTQ)	−0.452***	−0.482***	−0.432***	0.523***										
6. Physical Abuse (CTQ)	−0.358***	−0.363***	−0.358***	0.336***	0.494***									
7. Sexual Abuse^a^ (CTQ)	−0.210***	−0.162**	−0.181**	0.182**	0.126*	0.259***								
8. Emotional Neglect (CTQ)	−0.485***	−0.471***	−0.451***	0.533***	0.681***	0.374***	0.135*							
9. Physical Neglect (CTQ)	−0.392***	−0.384***	−0.396***	0.455***	0.578***	0.389***	0.192***	0.770***						
10. Childhood Trauma	−0.507***	−0.508***	−0.486***	0.564***	0.790***	0.511***	0.199***	0.940***	0.880***					
11. Care (PBI)	0.498***	0.500***	0.508***	−0.536***	−0.695***	−0.378***	−0.140*	−0.876***	−0.749***	−0.882***				
12. Overprotection (PBI)	−0.507***	−0.507***	−0.463***	0.488***	0.499***	0.399***	0.140*	0.481***	0.356***	0.511***	−0.486***			
13. Healthcare Utilization	−0.442***	−0.460***	−0.421***	0.367***	0.335***	0.235***	0.179**	0.334***	0.230***	0.344***	−0.331***	0.301***		
14. Morbidity	−0.447***	−0.445***	−0.441***	0.453***	0.361***	0.276***	0.076	0.360***	0.337***	0.389***	−0.367***	0.368***	0.386***	

*Note:* Spearman correlations were used due to non‐normal distributions across all variables (Shapiro–Wilk *p* < 0.001). Higher scores on Social Functioning indicate poorer functioning. Higher scores on CTQ subscales indicate greater trauma exposure. Higher PBI Care scores indicate greater perceived parental care; higher PBI Overprotection scores indicate greater perceived overprotection.

^a^ Sexual Abuse subscale reported for descriptive purposes only.

* *p* < 0.05.

** *p* < 0.01.

*** *p* < 0.001.

*Social functioning* was assessed using the Social Functioning Questionnaire [SFQ] (Tyrer et al. 2005), an 8‐item instrument evaluating perceived functioning across key life domains including work and household management, financial status, interpersonal and sexual relationships, family relationships, and leisure activities. Items are rated on a 4‐point scale (0–3), and the composite score is the sum of all items, yielding a range of 0–24, with higher scores indicating poorer social functioning. The SFQ has demonstrated acceptable internal consistency in previous research (*α* = 0.64; Oltmanns et al. 2002). In the present study, internal consistency was *α* = 0.835. As no validated Hebrew version existed at the time of data collection, a systematic translation‐retranslation procedure was applied and the resulting version was reviewed by linguistic and content experts prior to use.

*Parental bonding* was assessed using the Parental Bonding Instrument [PBI] (Parker et al. 1979), a 25‐item retrospective questionnaire measuring perceived childhood parental behavior across two subscales: Care (12 items) and Overprotection (13 items). Items are rated on a 4‐point scale, and subscale scores are the sum of their respective items. Higher Care scores reflect greater perceived parental warmth and involvement; higher Overprotection scores reflect greater perceived parental control and intrusiveness. The PBI has demonstrated good internal consistency in previous research (*α* = 0.83–0.94; Cohen and Finzi‐Dottan 2005; Weshler 2019). In the present study, internal consistency was high for both the Care subscale (*α* = 0.948) and the Overprotection subscale (*α* = 0.916). Participants were instructed to complete the PBI with specific reference to the parent with SMI.

*Physical and mental morbidity* was assessed using a 22‐item researcher‐developed checklist adapted from the EUROHIS survey instrument (Ministry of Health 2017). Participants indicated which of 22 listed acute and chronic medical and psychiatric conditions they had been diagnosed with; each endorsed item was scored as 1. The composite score is the sum of all endorsed items, with higher scores reflecting greater morbidity burden. Internal consistency was acceptable given the heterogeneous multi‐domain nature of the instrument (*α* = 0.709), consistent with expectations for checklists not designed to measure a unidimensional construct (Streiner 2003).

*Healthcare service utilization* was assessed using an 11‐item researcher‐developed instrument adapted from the EUROHIS survey tool (Ministry of Health 2017), supplemented with additional items specific to mental health service use including visits to psychiatrists or mental health professionals, in/outpatient mental health clinic attendance, and psychiatric inpatient admissions. Items capture utilization over the past 4 weeks (outpatient visits) and past year (hospitalizations). Two hospitalization items were recoded from a yes/no format to a quantitative variable capturing the number of episodes. The composite score is the sum of all items, with higher scores indicating greater healthcare utilization. Given the heterogeneous nature of the instrument spanning diverse service types: outpatient visits, specialist consultations, emergency care, and psychiatric admissions, low internal consistency is expected and does not indicate poor instrument quality. Healthcare utilization is not a unidimensional construct; individuals may access some service types while avoiding others based on need, access, and help‐seeking patterns, and a summed score reflects total utilization burden rather than a latent trait (Streiner 2003). This approach is consistent with standard practice in health services research, where heterogeneous utilization checklists are routinely analyzed as summed indices (Selden et al. 2024).

### 3.7 Data Analysis

All statistical analyses were conducted using IBM SPSS Statistics version 31 (IBM Corp. 2022). Internal consistency of all study instruments was evaluated using Cronbach's alpha. Normality was assessed using the Shapiro–Wilk test, along with visual inspection of histograms and skewness and kurtosis values. Given non‐normal distributions, Spearman rank‐order correlations were used for all bivariate analyses.

The analytical strategy proceeded in two stages. First, a comprehensive correlation matrix was computed to examine bivariate associations among study variables. Second, multivariate regression models were conducted to examine the unique contribution of each predictor while controlling for demographic covariates. Regression models were structured hierarchically, with demographic variables entered in the first block and study‐specific predictors in the second block.

Quality of life and social functioning were analyzed using hierarchical multiple linear regression. Morbidity and healthcare utilization, as overdispersed count variables with a high proportion of zero values, were analyzed using negative binomial regression with a log link function (Hilbe 2011). Birth order was recoded into four categories to ensure adequate cell sizes for stable parameter estimation. Model fit was evaluated using deviance and Pearson chi‐square statistics relative to degrees of freedom.

The moderating role of resilience in the relationship between childhood trauma and morbidity (Aim 3) and social functioning (Aim 4) was examined using the PROCESS macro, specifying Model 1. All variables were mean‐centered prior to computing interaction terms, and moderation was evaluated based on the interaction coefficient and its associated *p* value (*α* = 0.05).

Multicollinearity was assessed using variance inflation factors, with VIF values below 10 considered acceptable, consistent with established methodological guidelines (Kutner et al. 2004; Myers 1990). Residual normality and homoscedasticity were evaluated using normal P–P plots and standardized residual scatterplots. No missing data were present. Statistical significance was set at *p* < 0.05 for all analyses.

## 4 Results

### 4.1 Preliminary Analyses

Descriptive statistics and normality test results for all study variables are presented in Table 2. All instruments demonstrated acceptable to excellent internal consistency (Cronbach's *α* = 0.514–0.948). Shapiro–Wilk tests indicated that all study variables significantly deviated from normality (*p* < 0.001). Quality of life, resilience, and self‐efficacy scores showed negative skew consistent with ceiling effects, whereas morbidity and healthcare utilization demonstrated marked positive skew with a high proportion of zero values. Participant characteristics are presented in Table 1.

Multicollinearity diagnostics indicated acceptable VIF values for all predictors across Models 2–4 (all VIF < 5.0). In Model 1, resilience and self‐efficacy demonstrated borderline multicollinearity (VIF = 5.43 and 5.54, respectively), reflecting their strong intercorrelation (*ρ* = 0.84). Both predictors remained statistically significant and were retained in the final model.

Residual diagnostic plots indicated that assumptions of normality and homoscedasticity were adequately met for linear regression models. Residual patterns for morbidity and healthcare utilization were consistent with count data distributions.

### 4.2 Bivariate Correlations

Spearman correlations among all study variables are presented in Table 3. Quality of life was significantly and positively associated with resilience (*ρ* = 0.693, *p* < 0.001) and self‐efficacy (*ρ* = 0.651, *p* < 0.001) and negatively associated with social functioning (*ρ* = −0.666, *p* < 0.001), morbidity (*ρ* = −0.447, *p* < 0.001), and healthcare utilization (*ρ* = −0.442, *p* < 0.001). Childhood trauma exposure was positively associated with social functioning (*ρ* = 0.564, *p* < 0.001) and morbidity (*ρ* = 0.389, *p* < 0.001). Parental care was negatively associated with social functioning (*ρ* = −0.536, *p* < 0.001), while parental overprotection was positively associated with social functioning (*ρ* = 0.488, *p* < 0.001). Morbidity was positively associated with healthcare utilization (*ρ* = 0.386, *p* < 0.001). Resilience and self‐efficacy demonstrated a strong intercorrelation (*ρ* = 0.840, *p* < 0.001). The CTQ Sexual Abuse subscale was the only variable that did not significantly correlate with morbidity (*ρ* = 0.076, *p* = 0.188); all other bivariate associations were statistically significant (*p* < 0.001).

### 4.3 Moderation Analyses

The moderating role of resilience in the relationship between childhood trauma and outcomes was examined using PROCESS Model 1. For morbidity (Aim 3), both trauma (*b* = 0.043, *p* < 0.001) and resilience (*b* = −0.068, *p* < 0.001) were significant predictors; however, the interaction term was not significant (*b* = −0.001, *p* = 0.199, Δ*R* ^2^ = 0.004), indicating no moderating effect. Similarly, for social functioning (Aim 4), trauma (*b* = 0.105, *p* < 0.001) and resilience (*b* = −0.304, *p* < 0.001) were significant predictors, while the interaction term was not significant (*b* = 0.000, *p* = 0.924, Δ*R* ^2^ = 0.000).

These findings indicate that resilience did not moderate the relationship between childhood trauma and either outcome in the present sample. However, given the ceiling effects observed in resilience scores and the resulting restriction of range, statistical power to detect interaction effects may have been limited, and a buffering effect cannot be ruled out entirely. Nonetheless, in the present sample, trauma and resilience appear to exert independent main effects.

### 4.4 Predictors of Quality of Life

Hierarchical multiple linear regression was conducted to examine predictors of quality of life (Table 4; full model coefficients, including all demographic covariates, are presented in Table S1). Demographic variables entered in Block 1 accounted for 62.5% of the variance (*R* ^2^ = 0.625, *F*(12, 287) = 39.93, *p* < 0.001), with self‐rated current health (*β* = 0.601, *p* < 0.001) and self‐rated financial situation (*β* = 0.194, *p* < 0.001) emerging as significant predictors. The addition of self‐efficacy, resilience, and morbidity in Block 2 explained an additional 11.2% of variance (Δ*R* ^2^ = 0.112, *F*(3, 284) = 40.59, *p* < 0.001), yielding a total *R* ^2^ of 0.738. In the final model, self‐efficacy (*β* = 0.238, *p* < 0.001), resilience (*β* = 0.191, *p* = 0.008), and morbidity (*β* = −0.119, *p* = 0.005) each independently predicted quality of life after controlling for demographic variables. Self‐rated current health remained the strongest predictor (*β* = 0.358, *p* < 0.001).

**TABLE 4:** Hierarchical multiple linear regression predicting quality of life (*N* = 300).

Variable	*B*	SE	*β*	*t*	*p*	95% CI
Block 1: Demographic variables						
*R* ^2^ = 0.625, *F*(12, 287) = 39.93, *p* < 0.001						
Block 2: Study predictors						
Δ*R* ^2^ = 0.112, *F*(3, 284) = 40.59, *p* < 0.001; Total *R* ^2^ = 0.738						
General Self‐efficacy (NGSE)	5.741	1.710	0.238***	3.357	< 0.001	[2.375, 9.108]
Resilience (CD‐RISC‐10)	0.435	0.163	0.191**	2.665	0.008	[0.114, 0.757]
Morbidity	−1.130	0.400	−0.119**	−2.826	0.005	[−1.917, −0.343]

*Note:* Demographic variables entered in Block 1 included gender, birth year, years of education, employment status, marital status, number of children, number of siblings, birth order, frequency of financial problems, self‐rated financial situation, self‐rated current health, and parent with SMI. Only Block 2 predictors are presented individually. Higher morbidity scores indicate greater illness burden.

** *p* < 0.01.

*** *p* < 0.001.

### 4.5 Predictors of Social Functioning

Hierarchical multiple linear regression was conducted to examine predictors of social functioning (Table 5; full coefficients are presented in Table S2). Demographic variables entered in Block 1 accounted for 53.4% of the variance (*R* ^2^ = 0.534, *F*(12, 287) = 27.42, *p* < 0.001), with frequency of financial problems (*β* = 0.289, *p* < 0.001), self‐rated current health (*β* = −0.339, *p* < 0.001), and self‐rated financial situation (*β* = −0.222, *p* < 0.001) emerging as significant predictors. The addition of childhood trauma exposure and parental bonding subscales in Block 2 explained an additional 9.2% of variance (Δ*R* ^2^ = 0.092, *F*(3, 284) = 23.34, *p* < 0.001), yielding a total *R* ^2^ of 0.626. In the final model, childhood trauma (*β* = 0.309, *p* < 0.001) and parental overprotection (*β* = 0.171, *p* < 0.001) were significant predictors of social functioning. Parental care was not a significant predictor (*β* = 0.051, *p* = 0.504), indicating that it did not contribute unique variance beyond the other variables included in the model.

**TABLE 5:** Hierarchical multiple linear regression predicting social functioning (*N* = 300).

Variable	*B*	SE	*β*	*t*	*p*	95% CI
Block 1: Demographic variables						
*R* ^2^ = 0.534, *F*(12, 287) = 27.42, *p* < 0.001						
Block 2: Study predictors						
Δ*R* ^2^ = 0.092, *F*(3, 284) = 23.34, *p* < 0.001; Total *R* ^2^ = 0.626						
Childhood trauma burden	0.102	0.027	0.309***	3.830	< 0.001	[0.050, 0.154]
Care (PBI)	0.025	0.037	0.051	0.669	0.504	[−0.048, 0.097]
Overprotection (PBI)	0.093	0.027	0.171***	3.489	< 0.001	[0.041, 0.146]

*Note:* Demographic variables entered in Block 1 included: gender, birth year, years of education, employment status, marital status, number of children, number of siblings, birth order, frequency of financial problems, self‐rated financial situation, self‐rated current health, and parent with SMI. Only Block 2 predictors are presented individually. Higher social functioning scores indicate poorer functioning.

*** *p* < 0.001.

### 4.6 Predictors of Morbidity

Given the overdispersed count nature of the morbidity outcome with a high proportion of zero values, negative binomial regression was employed (Table 6; full coefficients are presented in Table S3). The overall model was statistically significant (*χ* ^2^(25) = 184.17, *p* < 0.001), with acceptable model fit (deviance/df = 1.18, Pearson *χ* ^2^/df = 1.12). Childhood trauma significantly predicted morbidity (*B* = 0.013, Exp(*B*) = 1.013, *p* = 0.002), indicating that each unit increase in trauma exposure was associated with a 1.3% increase in the expected morbidity count, controlling for demographic variables. Self‐rated current health was the strongest predictor (Wald *χ* ^2^ = 32.25, *p* < 0.001), with each unit increase associated with a 19.3% decrease in expected morbidity (Exp(*B*) = 0.807). Year of birth also significantly predicted morbidity (*B* = −0.028, Exp(*B*) = 0.972, Wald *χ* ^2^(1) = 12.79, *p* < 0.001), indicating that older participants had higher expected morbidity counts, consistent with longer cumulative exposure to health risks.

**TABLE 6:** Negative binomial regression predicting morbidity (*N* = 300).

Variable	*B*	SE	Exp(*B*)	Wald *χ* ^2^	df	*p*	95% CI
Demographic variables (Block 1)							
*χ* ^2^(25) = 184.17, *p* < 0.001; Deviance/df = 1.181; Pearson *χ* ^2^/df = 1.123							
Study predictors							
Childhood trauma burden	0.013	0.004	1.013	9.886	1	0.002	[1.005, 1.022]
Resilience (CD‐RISC‐10)	−0.013	0.008	0.987	2.613	1	0.106	[0.973, 1.003]

*Note:* Negative binomial regression with log link was employed given the overdispersed count nature of the morbidity outcome, which included a high proportion of zero values. Demographic covariates included in the model are presented as a block summary only.

Resilience was not a significant predictor of morbidity after controlling for demographic variables (*B* = −0.013, Exp(*B*) = 0.987, *p* = 0.106).

### 4.7 Predictors of Healthcare Utilization

Negative binomial regression was employed to examine predictors of healthcare utilization (Table 7; full coefficients are presented in Table S4). The overall model was statistically significant (*χ* ^2^(24) = 119.74, *p* < 0.001), with acceptable model fit (deviance/df = 1.22, Pearson *χ* ^2^/df = 1.01).

**TABLE 7:** Negative binomial regression predicting healthcare utilization (*N* = 300).

Variable	*B*	SE	Exp (*B*)	Wald *χ* ^2^	df	*p*	95% CI
Demographic variables							
*χ* ^2^(24) = 119.74, *p* < 0.001; Deviance/df = 1.217; Pearson *χ* ^2^/df = 1.005							
Study predictors							
Morbidity	0.111	0.039	1.117	8.095	1	0.004	[1.036, 1.207]

*Note:* Negative binomial regression with log link was employed given the overdispersed count nature of the healthcare use outcome, which included a high proportion of zero values. Demographic covariates included in the model are presented as a block summary only.

Morbidity significantly predicted healthcare utilization (*B* = 0.111, Exp(*B*) = 1.117, *p* = 0.004), indicating that each additional morbidity condition was associated with an 11.7% increase in the expected healthcare utilization count, controlling for demographic variables. Self‐rated current health was the strongest predictor (Wald *χ* ^2^ = 22.02, *p* < 0.001), with each unit increase associated with an 18.7% decrease in expected healthcare utilization (Exp(*B*) = 0.813).

Years of formal education also significantly predicted healthcare utilization (*B* = 0.074, Exp(*B*) = 1.077, *p* = 0.004), indicating that each additional year of education was associated with a 7.7% increase in expected healthcare utilization.

Self‐rated financial situation negatively predicted healthcare utilization (*B* = −0.098, Exp(*B*) = 0.907, Wald *χ* ^2^(1) = 6.06, *p* = 0.014), indicating that participants reporting poorer financial situations accessed fewer healthcare services.

## 5 Discussion

This study examined risk and protective factors associated with quality of life, social functioning, morbidity, and healthcare utilization among 300 Israeli adults who grew up with a parent with SMI. The findings revealed a consistent pattern in which personal adaptive resources of resilience and self‐efficacy independently predict subjective wellbeing, while childhood adversity and parental behavior are associated with social and health outcomes across adulthood. Notably, resilience did not amplify or buffer the effects of childhood trauma, suggesting that in this population, personal resources and early adversity operate through distinct and parallel pathways rather than interacting ones.

### 5.1 Self‐Efficacy, Resilience, and Quality of Life

Both general self‐efficacy and personal resilience were significant independent predictors of quality of life in the final multivariate model, supporting Aims 1 and 2 of the study. These findings align with a substantial body of evidence demonstrating that internal personal resources are central to subjective wellbeing among individuals navigating the long‐term consequences of adverse developmental experiences (Bandura 1997; Leiting et al. 2024; Connor and Davidson 2003).

The fact that resilience showed a slightly stronger bivariate association with quality of life than self‐efficacy is theoretically consistent with conceptualizations of resilience as a dynamic adaptive process that is associated with one's capacity to sustain wellbeing across changing circumstances, whereas self‐efficacy reflects a more task‐specific belief in one's capabilities (Masten 2024; Schwarzer and Warner 2013). However, both constructs contributed independently to quality of life after controlling for all demographic variables, indicating that they capture meaningfully distinct aspects of personal adaptive functioning.

The strong intercorrelation between resilience and self‐efficacy observed in this sample (*ρ* = 0.840) is noteworthy and extends beyond statistical considerations related to multicollinearity. One possible interpretation is that adults who grew up with a parent with SMI develop these capacities in parallel as part of an adaptive response to chronic adversity (Shestiperov et al. 2024). In this context, resilience may reflect the capacity to endure and recover from hardship, whereas self‐efficacy reflects confidence in one's ability to influence outcomes (Bandura 1997; Masten 2024). Theoretically and empirically, these are distinct constructs, measured by separate validated instruments with different developmental origins and different intervention targets (Bandura 1997; Masten 2024).

The multicollinearity observed in the quality of life model (VIF = 5.43 and 5.54) was addressed through several analytical considerations. First, while these values exceed the conservative threshold of 5.0, they remain well below the critical threshold of 10.0 that is equally cited in the methodological literature as indicative of problematic multicollinearity (Kutner et al. 2004; Myers 1990), and tolerance values of approximately 0.18 are above the critical cutoff of 0.10. Second, both predictors remained independently statistically significant in the final model, the key empirical indicator that multicollinearity did not inflate standard errors to a degree that compromised inference. Third, sensitivity checks confirmed the robustness of each predictor independently: when entered alone with morbidity, resilience remained a highly significant predictor of quality of life (*β* = 0.387, *p* < 0.001), as did self‐efficacy when entered alone with morbidity (*β* = 0.392, *p* < 0.001; Table S5). The larger standardized coefficients observed in these separate models, relative to the full model (*β* = 0.191 and *β* = 0.238, respectively), are consistent with the expected redistribution of shared variance between two correlated predictors when entered together, rather than evidence that either predictor's association with quality of life is spurious. Fourth, the differential behavior of resilience and self‐efficacy across outcome domains in this study provides further evidence that they are not interchangeable: resilience was a significant predictor of quality of life but not of morbidity after demographic controls were applied, whereas self‐efficacy predicted quality of life. Had the two constructs been functionally redundant, their pattern of associations across outcomes would be identical. It is not. Running separate models for each predictor or collapsing them into a composite adaptive resources factor, while statistically defensible approaches, would obscure this theoretically and empirically meaningful distinction without corresponding gain in interpretive clarity.

These constructs may therefore represent closely related but distinct aspects of adaptive functioning that co‐develop over time in response to chronic adversity exposure. The strength of their association in this sample may reflect the cumulative impact of growing up in environments of sustained relational unpredictability, although this pattern warrants caution in interpretation. Future research should examine whether resilience and self‐efficacy function as distinct predictors across varying levels of adversity severity and in other populations.

### 5.2 Childhood Trauma, Parental Bonding, and Social Functioning

Childhood trauma exposure was a key predictor of social functioning in the multivariate model, consistent with extensive evidence linking early adversity to long‐term interpersonal and functional difficulties in adulthood (Daníelsdóttir et al. 2024; Fares‐Otero et al. 2023; Norman et al. 2012). For adults who grew up with a parent with SMI, chronic exposure to emotional neglect and relational unpredictability during childhood may fundamentally be associated with attachment patterns and interpersonal schemas in ways that persist into adulthood, creating enduring vulnerabilities in social functioning that demographic factors alone cannot explain (Murphy et al. 2015; Shestiperov et al. 2024).

Parental overprotection independently predicted poorer social functioning after controlling for trauma exposure and all demographic covariates, extending prior research on the developmental consequences of overcontrolling parenting in clinical populations (Bruysters and Pilkington 2023; de Roo et al. 2022; Parker et al. 1979). This finding suggests that the behavioral dimension of parental bonding, characterized by excessive control, intrusion, and fostering of dependence, carries consequences for adult social competence that are distinct from and in addition to those of direct traumatic exposure. In the context of parental SMI, overprotective parenting behaviors may reflect the parent's own anxiety or illness‐related difficulties rather than intentional parenting strategies. However, regardless of their origin, such behaviors may be associated with limited opportunities for autonomy and social development and with poorer social functioning in adulthood (Bruysters and Pilkington 2023; de Roo et al. 2022).

Notably, parental care lost statistical significance in the multivariate model after controlling for demographics, indicating that it did not contribute unique variance beyond other variables in the model. This finding may reflect the complex relational dynamics within families affected by parental SMI, where caregiving relationships are frequently disrupted, inconsistent, and complicated by role reversals and parentification, making the construct of parental care particularly difficult to separate from broader socioeconomic and family structural factors in this population (Campbell and Patrick 2023; Shestiperov et al. 2024).

### 5.3 Childhood Trauma and Morbidity

Childhood trauma significantly predicted morbidity in the negative binomial regression model, with each unit increase in trauma exposure associated with a 1.3% increase in expected morbidity count after controlling for demographic variables. While this effect size appears modest at the item level, it reflects a cumulative dose–response relationship across the full trauma exposure continuum, consistent with established evidence linking adverse childhood experiences to elevated physical and mental morbidity in adulthood through biological, psychological, and behavioral pathways (Senaratne et al. 2024; Dube et al. 2003; Shonkoff et al. 2012). Importantly, self‐rated current health emerged as the dominant predictor of morbidity across all analyses, reflecting the well‐established relationship between subjective health perception and objective health burden and suggesting that participants' self‐assessments were closely calibrated to their actual health status.

### 5.4 Morbidity, Quality of Life, and Healthcare Utilization

Morbidity significantly predicted both quality of life and healthcare utilization, consistent with the role of physical and mental health burden in linking early adversity to current functional outcomes in this population. The finding that each additional morbidity condition was associated with an 11.7% increase in expected healthcare utilization carries direct clinical significance, suggesting that effective identification and management of comorbid health conditions in adults who grew up with a parent with SMI may substantially reduce both individual suffering and healthcare system demands over time (Selden et al. 2024; Peterson et al. 2023).

The significant positive association between years of formal education and healthcare utilization, with more educated participants accessing more services, is consistent with health literacy research demonstrating that educational attainment facilitates healthcare navigation, symptom recognition, and help‐seeking behavior (Berkman et al. 2011; Gonçalves‐Fernández and Pino‐Juste 2025). Conversely, the negative association between self‐rated financial situation and utilization suggests that financial barriers limit healthcare access for lower‐income individuals within this already vulnerable population (Gunja et al. 2023). Taken together, these findings highlight the compounding disadvantage faced by adults with parental SMI backgrounds who also carry lower socioeconomic resources.

The association between morbidity and healthcare utilization, although statistically significant, was relatively modest in magnitude (Exp(*B*) = 1.117), suggesting that increased health burden is not proportionally associated with healthcare use in this population. Adults who grew up with parents with SMI have been characterized in qualitative research as demonstrating strong patterns of self‐reliance and reluctance to acknowledge vulnerability or seek external support, even when facing significant health difficulties (Salaheddin and Mason 2016; Shestiperov et al. 2024). These patterns of self‐reliance, developed in response to growing up in unpredictable family environments, may be associated with underutilization of healthcare services relative to their actual health needs. As a result, the morbidity burden in this population may be greater than what is reflected by healthcare utilization data alone. Nurses and other healthcare providers should be aware of this dynamic and consider proactive assessment and outreach strategies rather than relying solely on self‐initiated help‐seeking in this population.

### 5.5 The Non‐Significant Moderation Findings

Contrary to the hypothesized buffering role, resilience did not significantly moderate the relationship between childhood trauma and either morbidity or social functioning in the present sample, though the possibility of a modest buffering effect cannot be excluded given the statistical constraints discussed below. These findings suggest that in adults who grew up with a parent with SMI, personal resilience resources operate as independent contributors to wellbeing rather than as protective moderators of the long‐term effects of trauma. This pattern aligns with theoretical models positioning resilience and adversity as parallel rather than interacting forces, with each exerting independent influence on outcomes (Bonanno 2004; Bonanno and Diminich 2013).

A theoretically meaningful interpretation of these non‐significant findings draws on the specific developmental context of this population. Unlike acute or episodic trauma, growing up with a parent with SMI typically involves chronic, pervasive relational adversity spanning the entire developmental period, including emotional neglect, role reversal, parentification, and attachment disruption (Brummelhuis et al. 2025; Campbell and Patrick 2023; Shestiperov et al. 2024). The resilience that develops within this context may be fundamentally oriented toward enabling daily functioning and maintaining outward stability rather than toward buffering or attenuating the accumulating biological and social consequences of chronic adversity exposure (Jørgensen et al. 2021; Abate et al. 2024). From this perspective, resilience in this population serves to support quality of life and subjective wellbeing, domains where its effects are clearly demonstrated in this study, while the pathways through which chronic early adversity is associated with health difficulties and social functioning impairments may operate through biological and relational mechanisms that personal resilience resources do not readily intercept (Chen et al. 2024).

Additionally, the ceiling effects observed in resilience scores in the present sample, reflecting generally high resilience levels among this community‐based sample may have constrained statistical power to detect interaction effects even if modest moderation exists. This population characteristic, while reflective of the remarkable adaptive capacity of many adults who grew up with parents with SMI, simultaneously limits the variability needed to detect moderation effects, suggesting that studies recruiting from more clinically impaired subpopulations may yield different findings.

### 5.6 Resilience as a Promoter of Wellbeing but Not a Protector Against Biological and Social Adversity Sequelae

A key theoretical pattern emerging from the present data is the differential role of resilience across outcome domains. Resilience was a robust independent predictor of quality of life and showed strong bivariate associations with social functioning, yet lost statistical significance as a predictor of morbidity after demographic controls were applied. This differential pattern suggests that resilience operates most powerfully in domains that are related to subjective psychological experience, how one feels about life and how one navigates interpersonal functioning, while having a more limited influence on the biological consequences of chronic early adversity that manifest as diagnosable health conditions.

This distinction has important theoretical implications. The developmental literature suggests that chronic early adversity exposure produces lasting neurobiological changes, including dysregulation of the stress response system, elevated inflammatory markers, and altered neural development that represent relatively stable biological substrates not readily modifiable by psychological resources alone (Bush et al. 2026; Shonkoff et al. 2012). Resilience may protect the individual's subjective experience and social engagement while operating in a different domain than these biological processes, resulting in the observed pattern of strong wellbeing effects alongside limited health effects. This interpretation positions resilience as a psychosocial resource that is associated with how individuals experience and navigate life with adversity‐related health burdens rather than as a resource that prevents those burdens from developing.

## 6 Clinical Implications for Nursing

The present findings carry several concrete implications for nursing assessment and practice with adults who grew up with a parent with SMI. First, routine nursing assessment with this population should incorporate evaluation of both personal adaptive resources and adversity history, as these variables independently predict current health and functional status. Brief validated measures of resilience and self‐efficacy can be integrated into clinical encounters without imposing significant burden and may identify individuals who are functionally high‐performing yet carrying significant unacknowledged health and emotional needs.

Second, the finding that parental overprotection independently predicts social functioning difficulties in adult children highlights the importance of exploring relational history, not only traumatic exposure, in clinical encounters with this population. Understanding the specific quality of the parenting relationship, and not only its most adverse dimensions, may enrich clinical formulation and inform more targeted intervention planning. Importantly, this finding also carries implications for clinicians working directly with parents who have SMI. Overprotective parenting behaviors, characterized by excessive control, intrusion, and fostering of dependence, may reflect the parent's own anxiety or illness‐related difficulties and are amenable to clinical intervention. Mental health professionals working with parents who have SMI should incorporate assessment of parenting practices into routine care, with particular attention to overprotective dynamics, and consider family‐focused approaches that support both parental recovery and the developmental needs of children and adult children across the lifespan. The Prato Collaborative recommendations provide a practical framework for integrating such family‐sensitive practice into mental health services (Reupert et al. 2022). Third, given that resilience is strongly associated with quality of life but is not associated with morbidity, resilience‐focused nursing interventions should be positioned as strategies for enhancing subjective wellbeing and functional engagement rather than as substitutes for direct health monitoring and medical management. This population requires both strengths‐based approaches that build on their considerable adaptive resources and proactive health assessment that does not assume that high functioning equals good health. Importantly, these recommendations are consistent with what adults who grew up with a parent with mental illness have themselves identified as priorities. In a Delphi study engaging adult children, clinicians, and academics, mental health and self‐care emerged as the only content area endorsed with consensus across all expert groups, receiving the highest mean rating among adult children themselves (*M* = 4.78; Patrick et al. 2023). This convergence between clinician‐identified risk factors and lived‐experience priorities strengthens the case for dual‐focus nursing approaches that address both psychological wellbeing and physical health needs simultaneously, rather than treating adaptive functioning as evidence of low clinical need.

Fourth, the pattern of self‐reliance and help‐seeking reluctance in this population (Shestiperov et al. 2024) suggests that nurses should not interpret low healthcare utilization as indicative of low need. Proactive, relationship‐based outreach, rather than reliance on patient‐initiated contact, may be essential for identifying and addressing unmet health needs in individuals whose adaptive style is oriented toward independence and self‐management. The findings of Patrick et al. (2023) offer important guidance on how such outreach might be structured. Adult children in that study preferred individual therapy over group formats, favored programs promoted through community‐level channels such as community centers and medical clinics rather than psychiatric settings, and emphasized the need for a safe, non‐judgmental space with facilitators who combine mental health training with lived experience. Taken together, these preferences suggest that this population is not resistant to support, but rather requires it to be accessible, non‐stigmatizing, and relationally grounded. A trauma‐informed approach that acknowledges the specific relational history of this population may be particularly important for establishing the therapeutic relationships needed to facilitate genuine help‐seeking.

### 6.1 Limitations

This study has several limitations. The cross‐sectional design precludes causal inference, and the directionality of observed associations cannot be established. The convenience sampling approach resulted in a predominantly employed, married, and highly educated sample, which may reflect self‐selection among higher‐functioning individuals, which may limit generalizability to more severely affected adults who did not participate. All measures relied on self‐report, introducing potential recall bias particularly for retrospective assessments of childhood trauma and parental bonding. The morbidity and healthcare utilization checklists were researcher‐developed instruments with lower internal consistency coefficients, which may have reduced the strength of observed associations. Healthcare utilization, in particular, is a heterogeneous construct spanning multiple service domains, and future research should examine whether patterns of association differ across specific service types such as primary care, specialist, emergency, and mental health service use. The CTQ Sexual Abuse subscale demonstrated near‐zero variance in the present sample and was excluded from inferential analyses, limiting conclusions regarding this specific trauma type. Resilience and self‐efficacy demonstrated strong intercorrelation (*ρ* = 0.840), resulting in borderline multicollinearity in the quality of life model; however, both variables remained significant predictors.

## 7 Conclusions

This study provides quantitative evidence that among adults who grew up with a parent diagnosed with SMI, resilience and self‐efficacy were independently associated with better quality of life, childhood trauma and parental overprotection are independently associated with poorer social functioning, and morbidity burden is associated with healthcare utilization. Resilience did not significantly moderate the effects of childhood trauma on health or social functioning outcomes in the present sample, suggesting it functions primarily as an independent resource supporting subjective wellbeing, though the possibility of a modest buffering effect under conditions of greater resilience variability cannot be excluded. These findings underscore the importance of dual‐focus nursing approaches that build on the considerable adaptive resources of this population while simultaneously addressing their physical health needs proactively, recognizing that high functioning and high need are not mutually exclusive in adults who grew up in the enduring shadow of parental severe mental illness.

## 8 Clinical Resources

- What is serious mental illness? https://smiadviser.org/about/serious‐mental‐illness
- Severe mental illness (SMI) and physical health inequalities: briefing. https://www.gov.uk/government/publications/severe‐mental‐illness‐smi‐physical‐health‐inequalities/severe‐mental‐illness‐and‐physical‐health‐inequalities‐briefing
- Mental health of children and parents—a strong connection. https://www.cdc.gov/childrensmentalhealth/features/mental‐health‐children‐and‐parents.html

## Funding

Sigma Theta Tau International for Global Nursing Excellence—Small Grant Program. The President of the State of Israel National Research Grant for Academic Excellence and Innovation in Mental Health Services.

## Disclosure

The authors have nothing to report.

## Ethics Statement

Approval for this study was obtained from the Helsinki Ethics Board of the Barzilai Hospital Medical Center, Approval number: #BRZ‐0099‐22, and the Institutional Review Board of Ben Gurion University, Approval number: #45‐2022.

## Conflicts of Interest

The authors declare no conflicts of interest.

## Supporting information


**Table S1:** Hierarchical Multiple Linear Regression Predicting Quality of Life (*N* = 300).
**Table S2:** Hierarchical Multiple Linear Regression Predicting Social Functioning (*N* = 300).
**Table S3:** Negative Binomial Regression Predicting Morbidity (*N* = 300).
**Table S4:** Negative Binomial Regression Predicting Healthcare Utilization (*N* = 300).
**Table S5:** Sensitivity Analysis: Quality of Life Models With Resilience and Self‐Efficacy Entered Separately (*N* = 300).

## Data Availability

The data that support the findings of this study are available on request from the corresponding author. The data are not publicly available due to privacy or ethical restrictions.
