# Supplementary material for: Growing in the Shadow of Mental Illness: Quality of Life, Health Status, Social Functioning, and Health Care Utilization Among Adults to Parents With Severe Mental Illness
Source: J Nurs Scholarsh. 2026 Jul 26;58(4):e70118. doi: 10.1111/jnu.70118 (PMC13402340; doi:10.1111/jnu.70118)
Supplement: Supplementary file 1 — Table S1: Hierarchical Multiple Linear Regression Predicting Quality of Life (N = 300). Table S2: Hierarchical Multiple Linear Regression Predicting Social Functioning (N = 300). Table S3: Negative Binomial Regression Predicting Morbidity (N = 300). Table S4: Negative Binomial Regression Predicting Healthcare Utilization (N = 300). Table S5: Sensitivity Analysis: Quality of Life Models With Resilience and Self‐Efficacy Entered Separately (N = 300). [file JNU-58-0-s001.docx]

**TABLE S1** Hierarchical Multiple Linear Regression Predicting Quality of Life (N = 300)

| **Variable** | **B** | **SE** | **β** | **t** | **p** | **95% CI Lower** | **95% CI Upper** | **VIF** |
| --- | --- | --- | --- | --- | --- | --- | --- | --- |
| **Block 1: Demographic Variables — R² = .625, F(12, 287) = 39.93, p < .001** | | | | | | | | |
| (Constant) | -87.53 | 193.04 |  | -0.453 | .651 | -467.47 | 292.42 |  |
| Participant gender | -0.61 | 1.36 | -.017 | -0.452 | .652 | -3.28 | 2.06 | 1.03 |
| Year of birth | 0.05 | 0.10 | .025 | 0.541 | .589 | -0.14 | 0.24 | 1.70 |
| Years of formal education | -0.19 | 0.27 | -.030 | -0.708 | .479 | -0.72 | 0.34 | 1.37 |
| Employment status | -0.27 | 0.99 | -.011 | -0.275 | .784 | -2.21 | 1.67 | 1.13 |
| Marital status | 1.83 | 1.21 | .065 | 1.515 | .131 | -0.55 | 4.22 | 1.39 |
| Number of children | 0.65 | 0.57 | .055 | 1.128 | .260 | -0.48 | 1.78 | 1.80 |
| Number of siblings | -0.62 | 0.47 | -.063 | -1.334 | .183 | -1.54 | 0.30 | 1.69 |
| Birth order among siblings | -0.03 | 0.70 | -.002 | -0.037 | .970 | -1.41 | 1.36 | 1.62 |
| Frequency of financial problems | -2.06 | 1.02 | -.099 | -2.016 | .045* | -4.08 | -0.05 | 1.85 |
| Self-rated financial situation | 1.70 | 0.44 | .194 | 3.869 | < .001*** | 0.83 | 2.56 | 1.92 |
| Self-rated current health | 6.06 | 0.45 | .601 | 13.373 | < .001*** | 5.17 | 6.96 | 1.55 |
| Parent with SMI | -0.10 | 0.70 | -.005 | -0.139 | .890 | -1.47 | 1.27 | 1.03 |
| **Block 2: Study Predictors — ΔR² = .112, F(3, 284) = 40.59, p < .001; Total R² = .738** | | | | | | | | |
| (Constant) | 161.61 | 169.03 |  | 0.956 | .340 | -171.10 | 494.33 |  |
| Participant gender | -0.64 | 1.14 | -.017 | -0.560 | .576 | -2.89 | 1.61 | 1.04 |
| Year of birth | -0.08 | 0.09 | -.038 | -0.936 | .350 | -0.25 | 0.09 | 1.83 |
| Years of formal education | -0.12 | 0.23 | -.018 | -0.512 | .609 | -0.56 | 0.33 | 1.37 |
| Employment status | 0.12 | 0.83 | .005 | 0.148 | .882 | -1.52 | 1.77 | 1.15 |
| Marital status | 1.48 | 1.02 | .052 | 1.454 | .147 | -0.52 | 3.49 | 1.40 |
| Number of children | 0.52 | 0.48 | .044 | 1.075 | .283 | -0.43 | 1.47 | 1.80 |
| Number of siblings | -0.39 | 0.39 | -.039 | -0.997 | .320 | -1.16 | 0.38 | 1.69 |
| Birth order among siblings | 0.09 | 0.59 | .006 | 0.154 | .878 | -1.07 | 1.26 | 1.63 |
| Frequency of financial problems | -0.88 | 0.88 | -.042 | -0.994 | .321 | -2.61 | 0.86 | 1.94 |
| Self-rated financial situation | 0.73 | 0.38 | .084 | 1.921 | .056 | -0.02 | 1.48 | 2.05 |
| Self-rated current health | 3.61 | 0.46 | .358 | 7.837 | < .001*** | 2.71 | 4.52 | 2.26 |
| Parent with SMI | 0.37 | 0.59 | .019 | 0.621 | .535 | -0.79 | 1.53 | 1.05 |
| General self-efficacy (NGSE) | 5.74 | 1.71 | .238 | 3.357 | < .001*** | 2.37 | 9.11 | 5.43 |
| Resilience (CD-RISC-10) | 0.44 | 0.16 | .191 | 2.665 | .008** | 0.11 | 0.76 | 5.54 |
| Morbidity | -1.13 | 0.40 | -.119 | -2.826 | .005** | -1.92 | -0.34 | 1.91 |
| *Note. Higher morbidity scores indicate greater illness burden. Reference categories for categorical covariates: female (gender); other (employment); other (marital status). * p < .05. ** p < .01. *** p < .001.* | | | | | | | | |

**TABLE S2** Hierarchical Multiple Linear Regression Predicting Social Functioning (N = 300)

| **Variable** | **B** | **SE** | **β** | **t** | **p** | **95% CI Lower** | **95% CI Upper** | **VIF** |
| --- | --- | --- | --- | --- | --- | --- | --- | --- |
| **Block 1: Demographic Variables — R² = .534, F(12, 287) = 27.42, p < .001** | | | | | | | | |
| (Constant) | 115.55 | 54.53 |  | 2.119 | .035* | 8.22 | 222.89 |  |
| Participant gender | -0.17 | 0.38 | -.018 | -0.449 | .654 | -0.93 | 0.58 | 1.03 |
| Year of birth | -0.05 | 0.03 | -.096 | -1.825 | .069 | -0.10 | 0.004 | 1.70 |
| Years of formal education | 0.02 | 0.08 | .012 | 0.263 | .792 | -0.13 | 0.17 | 1.37 |
| Employment status | -0.13 | 0.28 | -.020 | -0.455 | .649 | -0.68 | 0.42 | 1.13 |
| Marital status | -0.60 | 0.34 | -.083 | -1.753 | .081 | -1.27 | 0.07 | 1.39 |
| Number of children | -0.22 | 0.16 | -.074 | -1.364 | .174 | -0.54 | 0.10 | 1.80 |
| Number of siblings | 0.14 | 0.13 | .057 | 1.087 | .278 | -0.12 | 0.40 | 1.69 |
| Birth order among siblings | 0.04 | 0.20 | .009 | 0.183 | .855 | -0.36 | 0.43 | 1.62 |
| Frequency of financial problems | 1.53 | 0.29 | .289 | 5.288 | < .001*** | 0.96 | 2.10 | 1.85 |
| Self-rated financial situation | -0.49 | 0.12 | -.222 | -3.968 | < .001*** | -0.74 | -0.25 | 1.92 |
| Self-rated current health | -0.87 | 0.13 | -.339 | -6.757 | < .001*** | -1.12 | -0.61 | 1.55 |
| Parent with SMI | -0.01 | 0.20 | -.002 | -0.043 | .966 | -0.40 | 0.38 | 1.03 |
| **Block 2: Study Predictors — ΔR² = .092, F(3, 284) = 23.34, p < .001; Total R² = .626** | | | | | | | | |
| (Constant) | 73.62 | 49.53 |  | 1.486 | .138 | -23.88 | 171.12 |  |
| Participant gender | -0.26 | 0.35 | -.028 | -0.751 | .453 | -0.94 | 0.42 | 1.04 |
| Year of birth | -0.03 | 0.02 | -.066 | -1.383 | .168 | -0.08 | 0.01 | 1.71 |
| Years of formal education | 0.06 | 0.07 | .034 | 0.808 | .419 | -0.08 | 0.19 | 1.38 |
| Employment status | 0.03 | 0.25 | .004 | 0.101 | .919 | -0.47 | 0.52 | 1.15 |
| Marital status | -0.51 | 0.31 | -.071 | -1.658 | .098 | -1.12 | 0.10 | 1.41 |
| Number of children | -0.19 | 0.15 | -.062 | -1.272 | .205 | -0.48 | 0.10 | 1.82 |
| Number of siblings | -0.02 | 0.12 | -.006 | -0.127 | .899 | -0.25 | 0.22 | 1.74 |
| Birth order among siblings | 0.10 | 0.18 | .027 | 0.578 | .564 | -0.25 | 0.46 | 1.63 |
| Frequency of financial problems | 1.34 | 0.26 | .254 | 5.102 | < .001*** | 0.82 | 1.86 | 1.88 |
| Self-rated financial situation | -0.34 | 0.11 | -.152 | -2.965 | .003** | -0.56 | -0.11 | 1.99 |
| Self-rated current health | -0.48 | 0.13 | -.187 | -3.826 | < .001*** | -0.72 | -0.23 | 1.82 |
| Parent with SMI | -0.18 | 0.19 | -.038 | -0.994 | .321 | -0.55 | 0.18 | 1.13 |
| Childhood trauma (CTQ) | 0.10 | 0.03 | .309 | 3.830 | < .001*** | 0.05 | 0.15 | 4.94 |
| Parental care (PBI) | 0.02 | 0.04 | .051 | 0.669 | .504 | -0.05 | 0.10 | 4.34 |
| Parental overprotection (PBI) | 0.09 | 0.03 | .171 | 3.489 | < .001*** | 0.04 | 0.15 | 1.83 |
| *Note. Higher social functioning scores indicate poorer functioning. Reference categories: female (gender); other (employment); other (marital status). * p < .05. ** p < .01. *** p < .001.* | | | | | | | | |

**TABLE S3** Negative Binomial Regression Predicting Morbidity (N = 300)

| **Parameter** | **B** | **SE** | **Exp(B)** | **Wald χ²** | **df** | **p** | **95% CI Lower** | **95% CI Upper** |
| --- | --- | --- | --- | --- | --- | --- | --- | --- |
| **Model fit: χ²(25) = 184.17, p < .001; Deviance/df = 1.18; Pearson χ²/df = 1.12; Negative binomial dispersion = 0.064 (SE = 0.057)** | | | | | | | | |
| (Intercept) | 56.88 | 15.65 | — | 13.20 | 1 | < .001*** | 26.20 | 87.55 |
| [Parent with SMI = Biological mother] | -0.13 | 0.62 | 0.880 | 0.043 | 1 | .835 | -1.34 | 1.08 |
| [Parent with SMI = Stepmother/foster] | 0.79 | 0.72 | 2.197 | 1.211 | 1 | .271 | -0.62 | 2.19 |
| [Parent with SMI = Biological father] | -0.28 | 0.62 | 0.752 | 0.212 | 1 | .645 | -1.50 | 0.93 |
| [Parent with SMI = Stepfather/foster] | 0 | — | 1 | — | — | — | — | — |
| [Gender = Male] | -0.01 | 0.12 | 0.986 | 0.015 | 1 | .902 | -0.24 | 0.21 |
| [Gender = Female] | 0 | — | 1 | — | — | — | — | — |
| [Employment = Unemployed] | -0.11 | 0.40 | 0.893 | 0.081 | 1 | .777 | -0.90 | 0.67 |
| [Employment = Pensioner] | 0.82 | 0.41 | 2.271 | 4.028 | 1 | .045* | 0.02 | 1.62 |
| [Employment = Self-employed] | 0.20 | 0.36 | 1.220 | 0.302 | 1 | .583 | -0.51 | 0.91 |
| [Employment = Employee] | 0.47 | 0.31 | 1.605 | 2.406 | 1 | .121 | -0.12 | 1.07 |
| [Employment = Other] | 0 | — | 1 | — | — | — | — | — |
| [Marital = Single] | 0.09 | 0.79 | 1.095 | 0.013 | 1 | .908 | -1.45 | 1.63 |
| [Marital = Married] | 0.04 | 0.81 | 1.046 | 0.003 | 1 | .956 | -1.54 | 1.63 |
| [Marital = Divorced] | -0.30 | 0.81 | 0.741 | 0.135 | 1 | .713 | -1.89 | 1.30 |
| [Marital = Other] | 0 | — | 1 | — | — | — | — | — |
| [Birth order = 1st] | -0.02 | 0.22 | 0.979 | 0.010 | 1 | .921 | -0.45 | 0.40 |
| [Birth order = 2nd] | 0.26 | 0.21 | 1.298 | 1.568 | 1 | .210 | -0.15 | 0.67 |
| [Birth order = 3rd] | 0.07 | 0.23 | 1.068 | 0.079 | 1 | .778 | -0.39 | 0.53 |
| [Birth order = 4th+] | 0 | — | 1 | — | — | — | — | — |
| [Financial problems = Very rarely] | -0.50 | 0.26 | 0.607 | 3.707 | 1 | .054 | -1.01 | 0.01 |
| [Financial problems = Rarely] | -0.25 | 0.25 | 0.779 | 0.965 | 1 | .326 | -0.75 | 0.25 |
| [Financial problems = Often] | -0.04 | 0.23 | 0.961 | 0.029 | 1 | .865 | -0.50 | 0.42 |
| [Financial problems = Very often] | 0 | — | 1 | — | — | — | — | — |
| Year of birth | -0.028 | 0.008 | 0.972 | 12.79 | 1 | < .001*** | -0.043 | -0.013 |
| Years of formal education | 0.035 | 0.024 | 1.036 | 2.161 | 1 | .142 | -0.012 | 0.081 |
| Number of children | 0.013 | 0.054 | 1.013 | 0.058 | 1 | .810 | -0.093 | 0.119 |
| Number of siblings | -0.011 | 0.041 | 0.989 | 0.069 | 1 | .793 | -0.090 | 0.069 |
| Self-rated financial situation | -0.034 | 0.036 | 0.966 | 0.914 | 1 | .339 | -0.104 | 0.036 |
| Self-rated current health | -0.215 | 0.038 | 0.807 | 32.25 | 1 | < .001*** | -0.289 | -0.141 |
| Resilience (CD-RISC-10) | -0.013 | 0.008 | 0.987 | 2.613 | 1 | .106 | -0.028 | 0.003 |
| Childhood trauma (CTQ) | 0.013 | 0.004 | 1.013 | 9.886 | 1 | .002** | 0.005 | 0.022 |
| (Scale) | 1 | — | — | — | — | — | — | — |
| (Negative binomial) | 0.064 | 0.057 | — | — | — | — | 0.011 | 0.365 |
| *Note. * p < .05. ** p < .01. *** p < .001.* | | | | | | | | |

**TABLE S4** Negative Binomial Regression Predicting Healthcare Utilization (N = 300)

| **Parameter** | **B** | **SE** | **Exp(B)** | **Wald χ²** | **df** | **p** | **95% CI Lower** | **95% CI Upper** |
| --- | --- | --- | --- | --- | --- | --- | --- | --- |
| **Model fit: χ²(24) = 119.74, p < .001; Deviance/df = 1.22; Pearson χ²/df = 1.01; Negative binomial dispersion = 0.456 (SE = 0.090)** | | | | | | | | |
| (Intercept) | -10.04 | 19.55 | — | 0.264 | 1 | .607 | -48.36 | 28.27 |
| [Parent with SMI = Biological mother] | -0.36 | 0.51 | 0.700 | 0.490 | 1 | .484 | -1.36 | 0.64 |
| [Parent with SMI = Stepmother/foster] | 0.40 | 0.73 | 1.485 | 0.295 | 1 | .587 | -1.03 | 1.82 |
| [Parent with SMI = Biological father] | -0.36 | 0.51 | 0.697 | 0.495 | 1 | .482 | -1.37 | 0.64 |
| [Parent with SMI = Stepfather/foster] | 0 | — | 1 | — | — | — | — | — |
| [Gender = Male] | -0.18 | 0.13 | 0.837 | 2.019 | 1 | .155 | -0.42 | 0.07 |
| [Gender = Female] | 0 | — | 1 | — | — | — | — | — |
| [Employment = Unemployed] | 0.55 | 0.44 | 1.738 | 1.609 | 1 | .205 | -0.30 | 1.41 |
| [Employment = Pensioner] | 0.55 | 0.48 | 1.736 | 1.313 | 1 | .252 | -0.39 | 1.49 |
| [Employment = Self-employed] | 0.72 | 0.36 | 2.046 | 4.040 | 1 | .044* | 0.02 | 1.41 |
| [Employment = Employee] | 0.42 | 0.31 | 1.519 | 1.815 | 1 | .178 | -0.19 | 1.03 |
| [Employment = Other] | 0 | — | 1 | — | — | — | — | — |
| [Marital = Single] | -0.47 | 0.70 | 0.628 | 0.449 | 1 | .503 | -1.83 | 0.90 |
| [Marital = Married] | -0.58 | 0.72 | 0.559 | 0.653 | 1 | .419 | -1.99 | 0.83 |
| [Marital = Divorced] | -1.00 | 0.74 | 0.368 | 1.840 | 1 | .175 | -2.45 | 0.44 |
| [Marital = Other] | 0 | — | 1 | — | — | — | — | — |
| [Birth order = 1st] | 0.05 | 0.24 | 1.056 | 0.053 | 1 | .818 | -0.41 | 0.52 |
| [Birth order = 2nd] | 0.24 | 0.23 | 1.268 | 1.087 | 1 | .297 | -0.21 | 0.68 |
| [Birth order = 3rd] | 0.10 | 0.25 | 1.107 | 0.168 | 1 | .682 | -0.39 | 0.59 |
| [Birth order = 4th+] | 0 | — | 1 | — | — | — | — | — |
| [Financial problems = Very rarely] | -0.16 | 0.31 | 0.855 | 0.250 | 1 | .617 | -0.77 | 0.46 |
| [Financial problems = Rarely] | -0.07 | 0.30 | 0.934 | 0.052 | 1 | .819 | -0.65 | 0.52 |
| [Financial problems = Often] | -0.24 | 0.29 | 0.791 | 0.657 | 1 | .418 | -0.80 | 0.33 |
| [Financial problems = Very often] | 0 | — | 1 | — | — | — | — | — |
| Year of birth | 0.006 | 0.010 | 1.006 | 0.394 | 1 | .530 | -0.013 | 0.025 |
| Years of formal education | 0.074 | 0.026 | 1.077 | 8.329 | 1 | .004** | 0.024 | 0.124 |
| Number of children | -0.031 | 0.056 | 0.969 | 0.312 | 1 | .576 | -0.140 | 0.078 |
| Number of siblings | 0.084 | 0.044 | 1.088 | 3.745 | 1 | .053 | -0.001 | 0.170 |
| Self-rated financial situation | -0.098 | 0.040 | 0.907 | 6.064 | 1 | .014* | -0.175 | -0.020 |
| Self-rated current health | -0.207 | 0.044 | 0.813 | 22.02 | 1 | < .001*** | -0.293 | -0.120 |
| Morbidity total | 0.111 | 0.039 | 1.117 | 8.095 | 1 | .004** | 0.035 | 0.188 |
| (Scale) | 1 | — | — | — | — | — | — | — |
| (Negative binomial) | 0.456 | 0.090 | — | — | — | — | 0.310 | 0.671 |
| *Note. * p < .05. ** p < .01. *** p < .001.* | | | | | | | | |

**TABLE S5** Sensitivity Analysis: Quality of Life Models With Resilience and Self-Efficacy Entered Separately (N = 300)

| **Model A: Demographic Covariates + Resilience + Morbidity (Self-Efficacy Excluded)** | | | | | |
| --- | --- | --- | --- | --- | --- |
| **Variable** | **B** | **SE** | **β** | **t** | **p** |
| *ΔR² = .102, F(2, 285) = 53.33, p < .001* |  |  |  |  |  |
| Resilience | .884 | .096 | .387*** | 9.249 | < .001 |
| Morbidity | -1.270 | .405 | -.133** | -3.137 | .002 |
|  |  |  |  |  |  |
| **Model B: Demographic Covariates + Self-Efficacy + Morbidity (Resilience Excluded)** | | | | | |
| *ΔR² = .106, F(2, 285) = 56.13, p < .001* |  |  |  |  |  |
| Self-Efficacy | 9.472 | .994 | .392*** | 9.531 | < .001 |
| Morbidity | -1.132 | .404 | -.119** | -2.801 | .005 |
| **Note.** ** p < .01. *** p < .001. |  |  |  |  |  |
|  |  |  |  |  |  |
